# Linking Dynamic Phenotyping with Metabolite Analysis to Study Natural Variation in Drought Responses of *Brachypodium distachyon*

**DOI:** 10.3389/fpls.2016.01751

**Published:** 2016-11-29

**Authors:** Lorraine H. C. Fisher, Jiwan Han, Fiona M. K. Corke, Aderemi Akinyemi, Thomas Didion, Klaus K. Nielsen, John H. Doonan, Luis A. J. Mur, Maurice Bosch

**Affiliations:** ^1^Institute of Biological, Environmental and Rural Sciences, Aberystwyth UniversityAberystwyth, UK; ^2^The National Plant Phenomics Centre, Institute of Biological, Environmental and Rural Sciences, Aberystwyth UniversityAberystwyth, UK; ^3^DLF Seeds A/S, Store HeddingeDenmark

**Keywords:** *Brachypodium distachyon*, drought, grasses, hormones, metabolite profiling, natural variation, phenotyping, stress

## Abstract

Drought is an important environmental stress limiting the productivity of major crops worldwide. Understanding drought tolerance and possible mechanisms for improving drought resistance is therefore a prerequisite to develop drought-tolerant crops that produce significant yields with reduced amounts of water. *Brachypodium distachyon* (Brachypodium) is a key model species for cereals, forage grasses, and energy grasses. In this study, initial screening of a Brachypodium germplasm collection consisting of 138 different ecotypes exposed to progressive drought, highlighted the natural variation in morphology, biomass accumulation, and responses to drought stress. A core set of ten ecotypes, classified as being either tolerant, susceptible or intermediate, in response to drought stress, were exposed to mild or severe (respectively, 15 and 0% soil water content) drought stress and phenomic parameters linked to growth and color changes were assessed. When exposed to severe drought stress, phenotypic data and metabolite profiling combined with multivariate analysis revealed a remarkable consistency in separating the selected ecotypes into their different pre-defined drought tolerance groups. Increases in several metabolites, including for the phytohormones jasmonic acid and salicylic acid, and TCA-cycle intermediates, were positively correlated with biomass yield and with reduced yellow pixel counts; suggestive of delayed senescence, both key target traits for crop improvement to drought stress. While metabolite analysis also separated ecotypes into the distinct tolerance groupings after exposure to mild drought stress, similar analysis of the phenotypic data failed to do so, confirming the value of metabolomics to investigate early responses to drought stress. The results highlight the potential of combining the analyses of phenotypic and metabolic responses to identify key mechanisms and markers associated with drought tolerance in both the Brachypodium model plant as well as agronomically important crops.

## Introduction

Drought, the sub-optimal supply of water, is an important environmental stress that limits the productivity of major crops worldwide. Climate change models predict greater variability in rainfall patterns and increased periods of summer drought, posing serious risks for food production. The cereals, wheat, rice, and maize, provide fifty percent of human dietary energy supply and climate change is projected to negatively impact on their production (IPCC, 2014). Likewise, a decline in the productivity of cereal crops and forages will impact on animal feed supplies and therefore affect the amount and price of milk and meat available for human consumption (Wheeler and Reynolds, 2013). As the global population increases, and hence the demand for staple cereal crops, drought will have widespread implications both on the environment and related socio-economic factors, agronomy, employment, migration, food security, health, and mortality (Stanke et al., 2013). Understanding drought tolerance and possible mechanisms for improving drought resistance in agronomically important crops is therefore a prerequisite to develop drought-tolerant crops that produce significant yields with reduced amounts of water.

Drought affects morphological, physiological, biochemical, and molecular processes in plants, resulting in growth inhibition. The extent of these changes is dependent on time, stage, and intensity of the drought stress (Chaves et al., 2009). Drought tolerance is therefore considered a complex trait under polygenic control and involving complex morpho-physiological mechanisms. Progress in improving drought tolerance in cereal crops and forages has been hampered by the lack of genetic and genomic tools, their large, and often polyploid, genomes and, critically, their large physical size and relatively long life cycles. *Brachypodium distachyon* (Brachypodium) contains many of the desirable properties required for a model system for the monocotyledon grasses (Opanowicz et al., 2008) and a number of genetic and genomic tools have been developed (Mur et al., 2011), including the genome sequence of accession Bd21 (The International Brachypodium Initiative, 2010). This has positioned Brachypodium as a powerful model species to accelerate trait improvement in cereals, forages and biomass crops (Rancour et al., 2012; Slavov et al., 2013; Girin et al., 2014). The Bd21 accession has been the main focus for experiments focussing on molecular analysis of drought stress (Bertolini et al., 2013; Verelst et al., 2013). The exploitation of genetic diversity in wild relatives and diversity germplasm of crops species holds the key to improving important agronomic traits. However, the domestication of cereal and forage crops has resulted in a decrease in genetic diversity compared to their wild ancestors (Buckler et al., 2001), decreasing our capacity for trait improvement in response to environmental stress including drought. Brachypodium grows in a wide variety of habitats and was never domesticated. This provides an excellent opportunity to identify drought associated gene-trait associations and transfer this knowledge to improve drought associated agronomic traits in important food and forage crops. The natural variation in aboveground and belowground physiology upon drought induced stress has been described for Brachypodium germplasm collections containing mainly material originating from Turkey (Luo et al., 2011; Chochois et al., 2015).

Conventional methods to phenotype drought related traits are time-consuming, labor intense, low throughput and often involve destructive harvest of plants making repeated measurements on the same plant impossible. Dynamic plant phenotyping methods enable controlled irrigation combined with automated imaging. These are particularly suited for studies on drought tolerance as these require accurate watering regimes and monitoring of responses over time to dissect the dynamic nature of drought development and the resulting stress response (Berger et al., 2010; Chen et al., 2014; Honsdorf et al., 2014).

While such phenotyping facilities have gained much interest in the context of increasing our understanding of the genetics of drought tolerance and for making gene-trait associations, the advantages it offers for metabolite–phenotype associations have yet to be explored. This is the case even though metabolite levels can be more closely linked to the macroscopic phenotype than genes as they reflect the integration of gene expression, enzyme activity, and other processes (Arbona et al., 2013) and, as such, are often used as predictive biomarkers. While metabolite profiling, as a diagnostic and predictive tool to identify novel markers for diseases, is widely used in the medical field, the application of biomarkers is not a common strategy to assist crop improvement (Steinfath et al., 2010). Drought stress leads to the accumulation of metabolites that function as osmolytes, antioxidants, or scavengers that help plants to avoid or tolerate stresses (Seki et al., 2007). Given the importance of drought induced biochemical changes, metabolic analyses provide a powerful approach to elucidate tolerance mechanisms and identify metabolic markers that may assist in developing drought-tolerant crops. For instance, a metabolomics study in oats (*Avena sativa*) has defined key processes involved in drought tolerance (Sanchez-Martin et al., 2015), while the combination of metabolite and phenotypic analysis identified correlations between certain metabolites and important fruit quality and yield traits in tomato (Schauer et al., 2006), highlighting the potential of metabolic markers for crop selection, evaluation and improvement.

In this study, we combine metabolic profiling with dynamic phenotyping of a diversity panel of Brachypodium accessions. We utilized an expanded germplasm collection of 138 Brachypodium diploid inbred lines (**Supplementary Table S1**) including ecotypes collected from a range of geographies and ecological niches from Northern Spain (Mur et al., 2011) to assess the natural variation in drought responses. Following initial drought screens, we used the facilities at the UK National Plant Phenomics Centre (NPPC-Aberystwyth) for high-resolution temporal imaging of 10 ecotypes with contrasting responses to drought stress to determine phenotypic trait associations to drought tolerance. Metabolite profiling of these samples not only informed on metabolic pathways involved in conferring drought tolerance in these selected Brachypodium ecotypes, but also enabled identification of key metabolite–phenotype associations. Our results identify several novel drought induced correlations between hormone pathways and phenotypic traits and provide a platform for improving our understanding of the genetics underlying drought associated metabolite–phenotype correlations essential for improving drought tolerance in cereal crops and forage grasses.

## Materials and Methods

### Plant Material

One hundred and thirty-eight Brachypodium diploid inbred lines were selected for the initial large drought screen, including ecotypes collected from a range of geographies and ecological niches (**Supplementary Table S1**), with 101 lines collected in Spain, 31 in Turkey, 3 in Iraq and one each from France, Italy, and Croatia.

### Initial Drought Screens

For the large drought screen, six replicates of 138 Brachypodium ecotypes were sown in small pots (7.5 cm diameter, 9 cm height) in 4:1 John Innes No 1 potting compost: grit mix. The pots were placed in a glasshouse at 21–22°C with 16 h of light (natural light supplemented with artificial light from 400-W sodium lamps). Two weeks after sowing plants were fully randomized. As it was our aim to induce drought stress in actively growing plants, well before they normally would start flowering, plants were not vernalized (this also applies to the second drought screen and the detailed plant phenotyping experiment). One ecotype, Arc23, was excluded as only three out of the six replicates had germinated. For the remaining 137 ecotypes, at least four replicate plants were available (114 with six replicates, 17 with five replicates, and 6 with four replicates). All seedlings were watered equal amounts prior treatment. Those replicates (2–3 plants per ecotype) to be exposed to water deficit were raised on circular blocks, approximately 1.5 cm in height, to prevent contact with any water draining through the soil of well-watered control plants. Total water withdrawal began 28 days after sowing and lasted 6 days. Leaf wilting was scored by two individuals according to a 1–6 scale for this trait (1 = no effect, 6 = severe effect) based on the visual assessment categories described by Engelbrecht et al. (2007). Plants were weighed immediately after harvest of the above ground biomass.

A second, refined drought screen was performed on 48 selected Brachypodium ecotypes (see **Supplementary Table S1**). Seedlings were germinated in square pots (9 cm × 9 cm × 7.5 cm), containing the same soil mix as before, in replicates of 12 (six controls and six to be stressed). Plants were grown under controlled environment conditions in a growth chamber at 21°C and under a 16 h photoperiod with 176 μmol m^-2^s^-1^ photon flux density supplied by white fluorescent tubes (OSRAM, Garching, Germany). All plants germinating within 1–2 days of each other (with the exceptions of ecotypes Kah6 and Uni14, which developed approximately 5 days later). Plants were randomized in a block design with buffer plants placed on outskirts to reduce changes in stress resulting from interactions with microclimate variations. Plants were all given equal volumes of water and water levels were monitored throughout, averaging at 0.25 m^3^ m^-3^ prior stress treatment and 0.29 m^3^ m^-3^ in soils of control plants during the stress treatment period. Total water withdrawal of six replicates per ecotype started 33 days after sowing and lasted for 12 days. The average soil moisture content upon drought treatment dropped to 0.024 m^3^ m^-3^ on day 42 while at the end of the experiment on day 45 no reading could be obtained.

Leaf wilting observations were taken after 12 days of drought treatment, using the same criteria as used in the large screen. On day 45, plants were harvested and fresh weight and dry weight biomass recorded; the latter after drying at 70°C for 3 days. Fresh and dry weight (DW) figures were then used to calculate above ground plant water content (PWC) on a fresh weight (FW) basis using the following equation: PWC (%) = [(FW_(g)_ - DW_(g)_)/FW_(g)_] × 100%.

### Phenotyping of Drought Induced Physiological Changes in Selected *Brachypodium* Ecotypes

Ten Brachypodium ecotypes were selected for detailed dynamic plant phenotyping using the facilities of the National Plant Phenomics Centre (NPPC) at Aberystwyth University. Seedlings were grown in 7.5 cm square pots, each containing 225 ± 0.5 g 4:1 Levington F2: grit sand in a regular glasshouse (for conditions see large drought screen). Twenty-eight days after sowing, the plants were transferred to the NPPC Smarthouse. Four plants of each ecotype were placed in a single tray lined with blue germination paper to ensure even distribution of water between the pots. Dividers were placed between the plants to enable imaging of individual plants, each of the trays was placed on a cart of the conveyer belt system (**Figure 2A**). All plants were watered at calculated 75% field capacity for an additional 2 days with the start of the treatments 33 days after sowing (das). Treatments were comprised of two levels of drought stress: 24 plants for each ecotype (six trays/carts) exposed to 15% soil water content (SWC), another set of 24 plants were not watered at all (0% SWC). Twelve control plants per ecotype (three trays/carts) continued to be watered to 75% SWC. Weighing and watering of the plants was fully automated. Samples of the compost were soaked to determine field capacity or dried to determine dry matter content. The water content was the difference between these two values. Target watering weight was calculated based upon a percentage of the water content. Plants from a parallel experiment were weighed at maximum size and this showed that plant fresh weight accounted for around 2% of the daily water usage, so no adjustment for plant biomass was deemed necessary. The different treatments lasted for 12 days with the aboveground biomass of four plants (one tray) per ecotype being harvested after 12 days for each of the treatments. Growth conditions in the Smarthouse were 22°C 16 h day/20°C night with supplementary top-up lighting. Watering was calculated based on the field capacity and dry matter content of the compost. Automated, target weight based watering was provided on a daily basis. Imaging was also performed daily starting from imposition of drought treatment (34 das) of all plants until the last day (45 das) of the treatments.

The timing for the initiation of the treatments for the two drought screens and for the phenotyping experiment (28, 33, and 33 das, respectively), as well as for the end-point of the treatments (34, 45, and 45 das, respectively), was based on our observations that Bd21, which does not require vernalization, started flowering ∼45 das. The chosen time-points therefore represented a reasonable equivalent for all ecotypes to be at a pre-flowering stage had they been vernalized.

### Feature Extraction

Images of each tray were taken from four side view angles with an interval of 90^o^ and one top view once a day. The camera used to image the plants exports 24 bit RGB color images, i.e., each channel has 256 class color levels. **Figure 2B** shows the position of four plants in each tray and the images acquired from side view and top view cameras. Images were processed to segment the plant from the background and to extract plant height, top view and side view projection area and color information. In order to acquire relatively uniform processing results for further analysis, images were filtered by a retina filter (Benoit et al., 2010), which enhances the contrast between plant and background, improves color consistency and provides spatial noise removal and luminance correction. Each side view image contains two individual plants, which are separated by a panel. Therefore after this pre-processing step, regions of interests (ROI) containing only one single plant were acquired with the bottom position of each ROI fixed just beyond the top of the pot from where the plant height and side view area are measured. **Figure 2C** illustrates the image processing on a side view image. In this image, the ROIs are fixed and height and total pixels (plant area) are acquired for each individual.

Within each ROI, plants were segmented according to the pixel colors in RGB color space. Although the pre-processing improved image quality and removed some noise, some small background pixel patches (clustering around 10–20 pixels) with the same colors of a plant, were removed by morphological operations.

Four features were extracted directly from the side view images of segmented plants: height, total pixels, yellow pixels, and grey pixels. The yellow color is defined as a color range where the red channel value is 10 levels higher than that of the green channel. The grey color range is defined by the following formula in which *V* denotes the value:

VGreenVBlue<2⁢ ⁢ and⁢ VGreenVBlue>1.15⁢ and⁢ VGreenVRed<1.4⁢ and⁢ ⁢ VGreen<150

The top view image processing extracted total pixel numbers for each of the four plants in a tray separated by two panels. For top view image processing, the color description of the plants needed to be more complex to extract as much as possible of the plant pixels while minimizing noise. **Figure 2D** shows the top view segmentation and pixel count. The colors of the plants were defined as:

VGreen<200⁢ and⁢ ⁢ VGreen>20⁢ and⁢ ⁢ VRed>20⁢ and⁢ ⁢ VRed<200and(VGreen−VBlue)>12⁢ and⁢ VRedVGreen<1.5and⁢   VGreenVBlue>1.5

All extracted values for height, estimated plant area and color information were written into a csv file for further analysis.

Image processing and feature extraction was achieved using C++ and OpenCV, an open source computer vision library^1^.

### Stomatal Conductance

Stomatal conductance was measured for 9 of the selected Brachypodium ecotypes (Bd21 was excluded as it started to flower during the course of the drought experiment) over a period of 7 days following drought treatment as well as for well-watered control plants at 5 weeks after sowing. Plants were grown under a 12 h light period (8 am – 4 pm) at 250 μmol m^-2^ s^-1^ photon flux density supplied by white fluorescent tubes (OSRAM) in a growth chamber at 20°C. Measurements of stomatal conductance were made on the second fully emerged true leaf between 12 noon and 1 pm using an AP4 porometer (Delta-T devices Ltd, Cambridge, UK).

### Metabolite Profiling

For each treatment (0%, 15%, and 75% SWC), leaf samples harvested 12 days after initiation of drought treatments, were pooled for each of the ecotypes and metabolites extracted following the procedure described by Allwood et al. (2006). Glass vials were capped and analyzed in random order on a LTQ linear ion trap (Thermo Electron Corporation). Data were acquired in alternating positive and negative ionization modes over four scan ranges (15–110, 100–220, 210–510, and 500–1200 *m/z*), with an acquisition time of 5 min. Discriminatory metabolites were selected and tentatively identified by following statistical analyses and interrogation of KEGG: Kyoto Encyclopedia of Genes and Genomes^2^ and MZedDB^3^. To substantiate these identifications, nominal mass signals were investigated further by targeted nano-flow Fourier Transform-Ion Cyclotron Resonance Ultra-Mass-Spectrometry (FT-ICR-MS) using TriVersa NanoMate (Advion BioSciences Ltd) on a LTQ-FT-ULTRA (Thermo Scientific) to obtain ultra-high accurate mass information and MSn ion-trees. Based on an accuracy of 1 ppm for the FT-ICR-MS, the top ranking metabolite with this range was indicated as the identification for each discriminatory negative ionization mode flow injection electrospray (FIE)-MS metabolite.

### Statistical Analysis

FIE-MS data was normalized with the total ion count for each sample used to transform the intensity value for each metabolite in to a percentage of the total ion count, after the removal of metabolites below 50 *m/z*. ANOVA, Pearsons correlation analyses, Principal Component Analyses (PCA), and Hierarchical Cluster Analyses (HCA) were completed using the R-based MetaboAnalyst 2.0 interface (Xia et al., 2015).

## Results

### Brachypodium Drought Screens

Two progressive drought screens were conducted with the aim to identify a core set of Brachypodium ecotypes for further detailed analysis. The initial large drought screen of 138 different Brachypodium ecotypes highlighted the considerable variation in the size and stature between the ecotypes, both in well-watered and drought stressed conditions. Wilting scores are the most widely used indicator for plant drought stress and have been shown to allow for robust ranking of survival when exposed to drought (Engelbrecht et al., 2007). Hence, visual assessment of plant wilting (**Supplementary Table S2**) formed the basis for the selection of those ecotypes to be included for a second, more refined drought screen. Other considerations taken into account for further selection were based on inclusion of ecotypes for resequencing^4^, available seed stock and developmental aspects.

**FIGURE 1:**
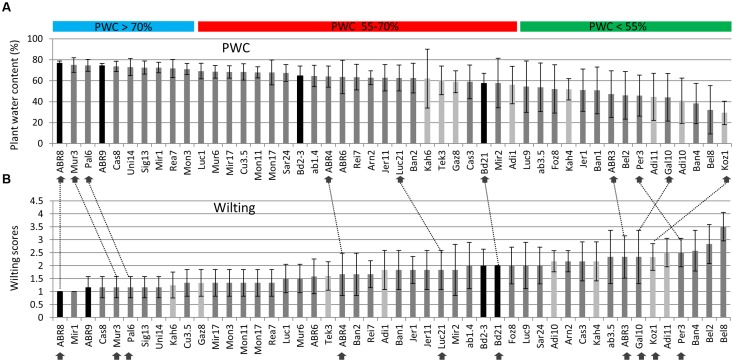
**Assessment of drought stress for selected Brachypodium ecotypes exposed to water deficit.** Plant water content (PWC) measures are ranked from high to low **(A)** and wilting scores from low (no sign of wilting) to high **(B)** for the 48 ecotypes included in the screen. Arrows indicate the ecotypes selected for further detailed analysis; dashed lines are included to compare the relative rankings of the selected ecotypes between **(A)** and **(B)**. Both PWC and wilting score data are based on six biological replicates, except for Foz8, Sar24, and Tek3 (five replicates) and Kah6 (four replicates). Origin: dark grey = Spain, light grey = Turkey, black bars = Other. Horizontal bars: PWC > 70% (Blue) = ecotypes classified as tolerant (TOL); PWC 55–70% (red) = ecotypes classified as intermediate (INT); PWC < 55% (green) = ecotypes classified as susceptible (SUS). Error bars indicate standard deviation.

A total of 48 different ecotypes were selected for the second drought screen, featuring bigger pots and controlled environment conditions as well as an increased number of replicates compared to the first screen. **Figure 1A** shows the distribution of the above ground plant water content (PWC) figures for the 48 ecotypes after withholding water for 12 days. During this period, the soil moisture content dropped from an average of ∼ 0.25 m^3^ m^-3^ before the treatment to ∼ 0.024 m^3^ m^-3^ 9 days after withholding water, while 12 days after treatment no readings could be obtained anymore. PWC ranged from just below 30% for Koz1 to almost 80% for ABR8. The ranking of the PWC was used as the main criterion for the selection of 10 ecotypes for further detailed analysis. Ecotypes ABR8, Pal6, and Mur3 were selected as drought resistant ecotypes as they showed the highest PWC upon drought treatment (**Figure 1A**). These ecotypes also showed a low wilting score in the second screen (**Figure 1B**). Koz1, Gal10, Per3 and ABR3 were selected as drought-susceptible ecotypes, since they featured amongst the lowest PWC scores and ranked highly in the wilting scores (**Figures 1A,B**). ABR4, Bd21, and Luc21 were included as having an intermediate (INT) response. As expected, comparison between the PWC data (**Figure 1A**) with the wilting scores (**Figure 1B**) shows that there is a strong negative correlation between these two measures (*r* = -0.869).

### Phenotypic Analyses of 10 Selected Brachypodium Lines

**FIGURE 2:**
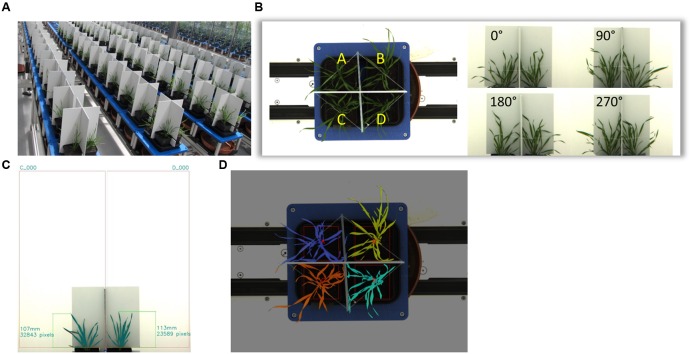
**Plant Phenotyping and image processing.** Setup of high-throughput phenotyping system. A total of 600 Brachypodium plants (10 genotypes) where phenotyped over 12 days **(A)**. Each tray holds four replicate individuals. The tray will be rotated four times with an angle of 90°. Therefore, each individual plant can have two side views from two orthogonal angles. The top view image is taken when the tray is at 0° **(B)**. The image processing is to segment the plant from background, such as the panels and wall. The program automatically finds the central separating panel to setup two regions of interests (ROI). The measurement happens in each ROI. The program copes with various lighting conditions and image quality **(C)**. The top view image processing finds the panels and uses them to separate those four plants in the tray. Then the total pixels of each plant are acquired. These plants are denoted by different colors **(D)**.

**FIGURE 3:**
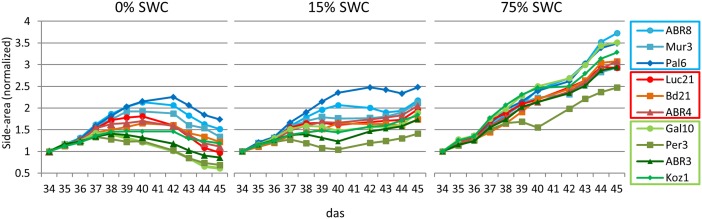
**Normalized shoot area curves.** Shoot areas for each of the 10 ecotypes for each treatment (0, 15, and 75% SWC) as extracted from RGB side images of the plants from day 34 to day 45 after sowing. Data was normalized against the values at 34 das (days after sowing). Error bars are not included to enable visual identification of the individual ecotypes (a list of normalized data including standard deviations are included in **Supplementary Table S3**. Blue colored lines = ecotypes classified as TOL; Red colored lines = ecotypes classified as INT; Green colored lines = ecotypes classified as susceptible (SUS).

Having selected 10 Brachypodium lines that were either classified as tolerant (TOL), INT, or susceptible (SUS) to the drought conditions imposed during the preceding drought screens (**Figure 1**); we next performed an integrated phenotype and metabolite analysis. Plants were exposed to two levels of drought targeting 15 and 0% SWC over a period of 12 days. RGB images were acquired daily from side views at different angles and top view and phenotypic features (height, area, and color) were extracted from the plant images (**Figure 2**). Plant area, as estimated from side view images, provides a proxy for growth (Neilson et al., 2015) and response to drought. An example of side-area over time for the 10 ecotypes is shown in **Figure 3**. Tolerant genotypes (blue lines) show higher accumulation of side area under water stress when compared to SUS (green) and INT (red) genotypes. Under well-watered control conditions there is no obvious distinction in side area accumulation according to the pre-classified tolerance groups (**Figure 3**). It should be noted that Bd21, included as an INT ecotype, started to flower during the course of the drought experiment and, therefore, was excluded from further phenotypic and metabolomic data analyses. To allow balanced multivariate analyses of our data, ABR3 was re-classified (as a line with borderline phenotype) and moved from the SUS to the INT drought tolerance group. The targeted genotypes were also assessed for stomatal performance under conditions tending toward 0% SWC (**Supplementary Figure S1**). Examination of stomatal performance under drought with each genotype and phenotypic class suggested that none exhibited significantly (*P* = 0.982) different responses. Thus, differential drought tolerance across the Brachypodium genotypes did not arise from stomatal effects.

**FIGURE 4:**
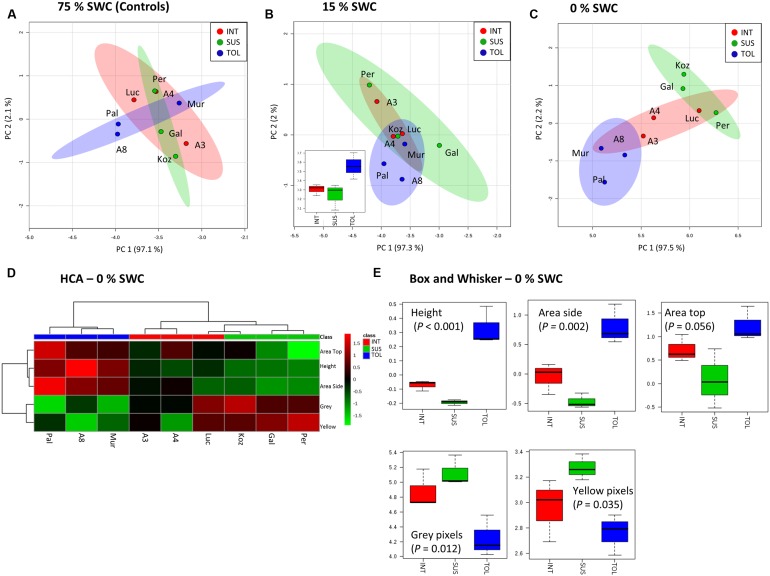
**Phenotypic descriptions of drought responses by Brachypodium accessions.** Principal Component Analyses of phenotypic data derived for conditions of **(A)** 75%, **(B)** 15%, and **(C)** 0% SWC. Data points are annotated according to genotypes and drought tolerance classes TOL (blue) = tolerant (Pal = Pal6; A8 = ABR8; Mur = Mur3); INT (red) = intermediate (Luc = Luc21; A3 = ABR3; A4 = ABR4) and SUS (green) = susceptible (Koz = Koz1; Gal = Gal10; Per = Per3). Larger circles indicate 95% confidence intervals for each phenotypic group that are appropriately color-coded. Inset within B is a box and whisker projection of the only statistically significant variable for 15% SWC conditions; “area side.” **(D)** Hierarchical cluster analyses (HCA) and **(E)** box and whisker projections of phenotypic data derived from 0% SWC condition. Levels of significance are indicated for each phenotypic character.

Unsupervised PCA was applied to the phenotypic data obtained at day 12 for the plants exposed to 15 and 0% SWC and compared to the fully watered 75% SWC controls. Data obtained from different genotypes were classified as TOL, INT, or SUS phenotypes based on our previous screens (**Figure 1**), and re-classification of ABR3 as mentioned before. PCA of phenotypic data obtained for 75% SWC controls suggest that most genotypes were morphologically similar with the possible exception of two TOL lines; Pal6 and ABR8 which lay outside the 95% confidence interval circles (**Figure 4A**). However, further analyses of these genotypes using ANOVA suggested that their most prominent feature (“area-side”) was not significantly different (*P* = 0.41) from the other genotypes. With imposition of drought to 15% SWC, phenotypic features were again not discriminating between the SUS and INT groups but were discriminating with two TOL genotypes Pal6 and ABR8 across PC2 (**Figure 4B**). ANOVA suggested that these two and indeed Mur3 were significantly taller (*P* = 0.04) as also indicated from box and whisker plots for plant height (**Figure 4B** inset). Considering phenotypic data for plants exposed to 0% SWC, clear differentiation was observed between TOL and SUS genotypes (**Figure 4C**). This aspect was also observed using HCA where each phenotypic group were clustered (**Figure 4D**). HCA and ANOVA (**Figure 4E**) demonstrated that drought tolerance was associated with significantly increased height and ‘area side’ but with reduced grey and yellow pixels.

### Metabolite Analyses Indicate Differential Responses to Drought within Each Phenotypic Group

**FIGURE 5:**
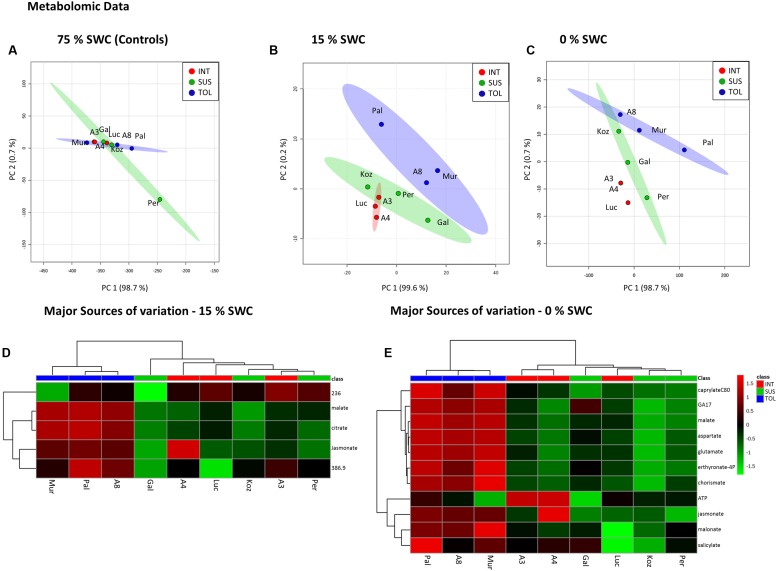
**Metabolomic responses to drought by Brachypodium accessions.** Principal Component Analyses of metabolomic data derived for conditions of **(A)** 75%, **(B)** 15%, and **(C)** 0% SWC. Data points are annotated according to genotypes and drought tolerance classes TOL (blue) = tolerant (Pal = Pal6; A8 = ABR8; Mur = Mur3); INT (red) = intermediate (Luc = Luc21; A3 = ABR3; A4 = ABR4) and SUS (green) = susceptible (Koz = Koz1; Gal = Gal10; Per = Per3). Larger circles indicate 95 % confidence intervals for each phenotypic group that are appropriately color-coded. Major source of variation in the datasets as defined by ANOVA for **(D)** 15% and **(E)** 0% SWC.

Metabolite analyses based on FIE-MS were performed on leaf samples collected 12 days after initiation of the two drought treatments and compared to well-watered controls. Images of the 9 genotypes recorded immediately before harvest for metabolite analysis are shown in **Supplementary Figure S2**. Non-supervised PCA of the well-watered control samples revealed no distinct clusters in the metabolite profiles of the three pre-defined phenotypic groups (**Figure 5A**). However, distinct clusters linked to relative drought tolerance emerge for the metabolites extracted from plants exposed to 15% SWC (**Figure 5B**), in particular for the TOL ecotypes. More severe drought to 0% SWC resulted in three clearly distinct clusters corresponding to TOL, SUS, and INT phenotypes (**Figure 5C**). The major source of variation were extracted for treatments to 15 and 0% SWC based on PCA loading vectors and significant differences as identified using ANOVA. These were tentatively identified by database interrogating based on high-resolution MS of the targeted *m/z.* For the 15% SWC samples the major sources of variation were tentatively associated with tricarboxylic acid (TCA) cycle intermediates malate and citrate, the phospholipid-derived hormone jasmonate, and unidentified metabolites with *m/z* of 236.01 and 386.9. HCA suggested that these metabolites alone could discriminate between the TOL and other groups at 15% SWC (**Figure 5D**). All with the exception of 236.01 *m/z* were relatively increased in the TOL grouping. Upon more severe drought (0% SWC) more metabolites were targeted which again could discriminate the TOL genotypes from the others (**Figure 5E**). These metabolites included jasmonate as well as other plant hormones gibberellin (GA17) and salicylate, metabolites associated with amino acid metabolism (aspartate, glutamate), chorismate, and lipid metabolism (caprylate). In each case, accumulation of the metabolites was relatively increased in the TOL lines.

**FIGURE 6:**
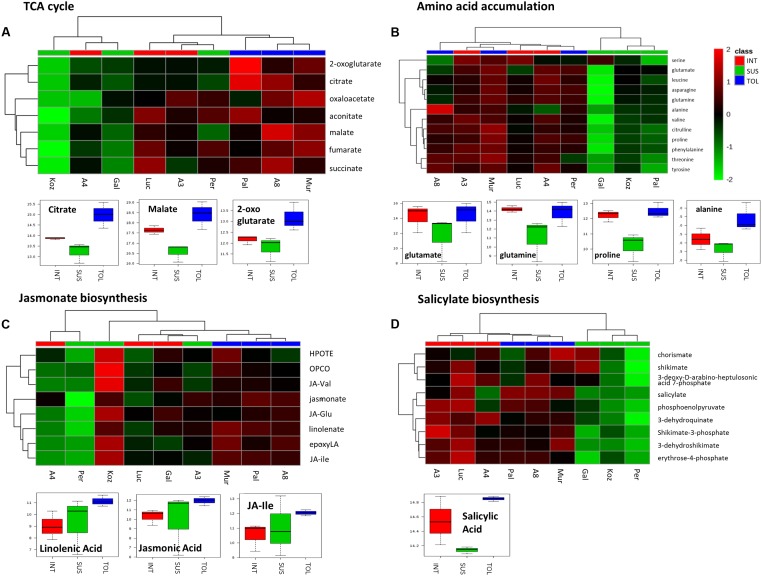
**Responses to drought by targeted biochemical pathways in Brachypodium accessions.** Previous analyses had targeted metabolites as major sources of variation. Mass-ions (*m*/z) tentatively linked to pathways where these key metabolites were components were extracted from the metabolomics data matrix. Metabolites tentatively linked to **(A)** the tricarboxylic acid (TCA) cycle, **(B)** amino acid accumulation, **(C)** jasmonate and **(D)** salicylate biosynthesis were compared by hierarchical cluster analyses. Metabolites in each pathway which exhibited significant changes between the TOL, INT and SUS phenotypic groups as defined by ANOVA are displayed using box and whisker plots.

To further highlight the possible importance of the pathways, *m/z* corresponding to metabolites within the pathways were extracted (**Figure 6**). In the case of the TCA cycle (**Figure 6A**), the putative TCA metabolites allowed the separate clustering of the TOL genotypes from other phenotypic classes. These data, as well as box and whisker plots, suggested significant increases in the TCA cycle in TOL genotypes. The TCA intermediates 2-oxoglutarate and oxaloacetate also contribute carbon skeletons for amino acid biosynthesis and examination of *m/z* tentatively linked to amino acid, suggested that lower levels of amino acid accumulation might be linked to a SUS phenotype (**Figure 6B**). Indeed, glutamate, glutamine, and the drought responsive amino acid proline were significantly elevated in both TOL and INT phenotypes. With alanine, significant increases were only associated with the TOL phenotypes.

Considering *m/z* tentatively linked with the biosynthesis of jasmonate or salicylate, HCA separated clustered TOL genotypes (**Figures 6C,D**). The TOL phenotypes exhibited significant increases in key oxylipins, linolenic acid, and the active jasmonate hormones, jasmonic acid (JA) and jasmonate-isoleucine (JA-Ile) (**Figure 6C**). With the salicylate pathway only salicylic acid (SA) itself (**Figure 6D**) was significantly increased in TOL phenotypes.

### Correlation between Metabolite and Phenotypic Traits

Since both the phenotypic data and the metabolite analyses reveal distinct clusters that match the designation of the different drought tolerance groups established during the initial screening, we wished to determine potential correlation between the metabolite pathway data and phenotypic variables. To assess this, a new matrix was derived which incorporated both metabolite and phenotypic data. Given the very different types of data, data was mean-centered and divided by the standard deviation of each variable. The data from the resulting matrix were established to be normally distributed.

The outcome of multivariate correlation analyses (based on Pearson’s *r* as a distance measure) is provided in **Supplementary Figure S3**. These analyses suggested that the putative jasmonate pathway correlated only with itself and no phenotypic measure. However, a key cluster (arrowed in **Supplementary Figure S3**) suggested positive correlations with phenotypic and metabolite indicators and a negative correlation with “Grey”/“Yellow” pixel counts.

**FIGURE 7:**
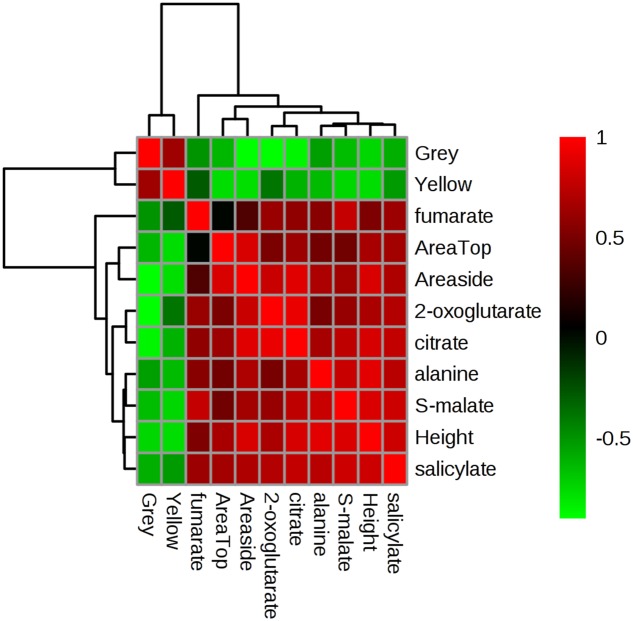
**Correlation analyses of phenotypic and metabolomic data.** Metabolites tentatively linked to four key pathways as targeted by unbiased chemometric analyses were correlated with phenotypic datasets (**Supplementary Figure S3**), based on which key relationships were identified. These form the basis of this Pearson’s correlation analyses the strength of which are indicated based on the intensity of red (positive) or green (negative) color. *r*^2^ values are provided in **Supplementary Table S4**.

To facilitate visualization of these trends, key variables in this cluster were extracted and separately analyzed (**Figure 7**). This highlighted that plant area (“top” and “side”) and height positively correlated with the relative concentrations of TCA intermediates, alanine and the hormone salicylate. This would suggest that tolerance in these Brachypodium genotypes was related to the accumulation of these metabolites. Linked to this, negative correlations were observed with grey and yellow pixel contents- features linked to susceptibility to drought.

## Discussion

The Poaceae (or grasses), including cereals and grasses of grasslands and pastures, constitute the major source of dietary calories for human and livestock, are increasingly important as lignocellulosic biomass for biofuels, and define many natural ecosystems and agricultural landscapes. Drought is an important environmental factor limiting the productivity of crops worldwide. The greater the diversity within a species, the better the potential for the species to overcome and adapt to changes in the environment, such as drought. Brachypodium displays considerable phenotypic variation across its geographic range, and is a key model species for cereals, forage grasses and energy grasses (Brkljacic et al., 2011). Its extensive natural variation encompasses many traits of agronomic or adaptive significance and, together with genetic and genomic tools available, is therefore well positioned for improving agronomic traits.

### Screening and Ecotype Selection

To assess the natural variation to drought stress and select ecotypes for further detailed studies, we performed two successive screens in which, respectively, 138 and 48 diploid Brachypodium ecotypes, sourced from different geographical locations, were exposed to progressive drought stress. These screens demonstrated the natural variation among Brachypodium ecotypes, in terms of morphology, biomass accumulation and response to drought stress. For instance, final dry weight biomass of the watered control replicates ranged from 0.032 to 0.317 g in the first screen and from 0.183 to 0.318 g in the second screen (data not shown). This was in line with the large phenotypic variation in response to drought stress observed amongst 57 Brachypodium ecotypes that mostly originated from Turkey (Luo et al., 2011).

When assessed for wilting, leaf water content (LWC) and chlorophyll fluorescence (*F*_v_/*F*_m_), these Brachypodium ecotypes were classified into four different groups ranging from TOL to most-SUS to drought stress (Luo et al., 2011). Assessment of genotypic variation has suggested greater genetic diversity in wild Brachypodium individuals from the Western Mediterranean region compared to those from the Eastern (Mur et al., 2011). Here we screened geographically more diverse ecotypes (101 lines collected in Spain, 31 in Turkey, 3 in Iraq, and 1 each from France, Italy, and Croatia), with our second screen only having six ecotypes (Adi10, Bd21, Koz1, Gaz8, Kah6, and Bd2-3) in common with the study by Luo et al. (2011).

Ecotypes were tentatively classified as TOL when exhibiting a PWC higher than 70% and SUS with a PWC lower than 55% with the ecotypes in-between classified as INT (**Figure 1A**). Brachypodium is found in many climate zones throughout temperate regions, spanning habitats near sea level to over 1800 m altitude (Des Marais and Juenger, 2016), suggesting that populations are locally adapted. It was therefore anticipated that similarly classified ecotypes might share similarities with regards to ecological niche. However, based on highland/lowland descriptions or altitude, such shared responses were not apparent in our screens. For example, two out of the three selected drought resistant ecotypes (Mur3 and Pal6) were from the highlands in Spain (ABR8 was from a hillside origin close to Siena in Italy), as were three of the four selected drought-susceptible ecotypes (ABR3, Per3 and Gal10; Koz1 is from the highlands in Turkey). A study looking at root phenotypes amongst 81 Brachypodium accessions also found no correlation between the phenotypic diversity and geographical origins (Chochois et al., 2015). This lack of correlation may suggest that the phenotypic plasticity of Brachypodium to changing environmental conditions (i.e., acclimation responses, Des Marais and Juenger, 2016) prevails over ecotypic differentiation (local adaptation, or within-species niche divergence leading to ecotypic differentiation, Liancourt and Tielbörger, 2009). It should be noted, however, that other features associated with ecological niches, such as soil type, acidity, and climate may still attribute to a shared drought response, but these parameters were not identified in this study.

### Phenotypical Characteristics of the Different Tolerance Groups

Maintaining plant growth and yield under drought represents a major objective for plant breeding. Growth, caused by cell division and cell elongation, is reduced under drought owing to impaired enzyme activities, loss of turgor, and decreased energy supply (Farooq et al., 2012). Most studies on drought are performed by withholding water, leading to progressive drought stress. However, under natural conditions plants are often exposed to moderate drought stress, in particular in temperate climates. As such, enhanced survival of *Arabidopsis thaliana* (Arabidopsis) under severe drought was shown not to be a good indicator for improved growth performance under mild drought conditions (Skirycz et al., 2011). In addition to progressive drought (0% SWC), we therefore exposed Brachypodium ecotypes to mild drought stress (15% SWC), utilizing the gravimetric watering control of the NPPC. Image based projected shoot area allows estimation of above ground biomass and, when assessed over time, can serve as a useful proxy for overall plant growth (Honsdorf et al., 2014; Neilson et al., 2015). On average, based on projected shoot area, exposure to moderate drought stress resulted in 85% more biomass accumulation during the treatment compared to exposure to severe stress (ranging from 43% for Pal6 to 211% for Gal10). Similarly, moderate drought imposed an average yield penalty of 38% (ranging from 28% for Mur3 to 46% for Gal10) when compared to well-watered controls. Results suggest that under mild drought stress, assessment of plant height, another parameter used to estimate plant biomass in several crop species (Tilly et al., 2015), could be a good predictor for ecotypes that perform well under more severe drought stress. However, the phenotypic features measured after exposure to moderate drought were unable to separate the Brachypodium ecotypes in the TOL, INT, and SUS groups.

In contrast to mild drought, PCA on the phenotypic data after progressive drought showed a clear differentiation between TOL and SUS genotypes. The TOL group was not only characterized by having significantly increased measures for height and side area, but also showed distinct color-related properties with significantly reduced proportions of yellow and grey pixels. While leaf ‘greenness’ has been shown to correlate with foliar nitrogen and chlorophyll, the relative proportion of yellow pixels is indicative of leaf senescence characterized by yellowing or chlorosis (Rajendran et al., 2009; Li et al., 2014; Neilson et al., 2015). Interestingly, we also identified a “grey” pixel color range that reflected the relationship between greenness and red and blue color pixels. The physical basis of this trait was not determined but could be due to altered pigmentation or water content. Thus, our results suggest reduced or delayed drought induced senescence in the TOL lines. Overall, our phenotypic data analyses upon exposure to progressive drought confirm the classification of the selected ecotypes into TOL, SUS, and INT, and therefore the robustness of the preceding drought screens.

### Metabolites Associated with Drought Stress

The interaction between genotype and environment is complex. The ability for metabolites to integrate these two components reflects an increasing tendency to use metabolites as selection markers in crop breeding programs to accelerate the development of improved cultivars tolerant to drought (Sanchez-Martin et al., 2015). Metabolites with a higher or indeed lower relative accumulation in the TOL lines when exposed to drought not only provide insights into the regulation of metabolic networks under drought stress, but could also lead to metabolite marker development. In this study, we used our well-established metabolomic analyses pipeline (e.g., Lloyd et al., 2011) to mine high-resolution FIE-MS datasets using multivariate approaches to identify major sources of variation in the experimental parameters. Through interrogation of the loading vectors linked to PCA, coupled with ANOVA of the datasets, we identified metabolites which exhibited differential accumulation in the TOL class in response to 0% SWC. Database interrogation with the highly resolved (to 1ppm) *m/z* suggested that these metabolites included three phytohormones, metabolites associated with amino acid metabolism and putative TCA cycle metabolites.

Phytohormones play critical roles in regulating plant responses to stress. Expression profiling of markers for defense-related phytohormones showed that Brachypodium has phytohormone responses more similar to those of rice than of Arabidopsis, suggesting that monocots share a common defense system that is different from that of dicots (Kouzai et al., 2016). Our analyses identified relative higher levels of GA, SA, and JA in the TOL ecotypes when exposed to severe drought with the latter also higher in TOL lines under mild drought. A central role for the GA class of growth hormones in the response to abiotic stress is becoming increasingly evident (Colebrook et al., 2014). For instance exogenous application of GA can alleviate drought-imposed adverse effects in maize (Akter et al., 2014), although application of GA has also been shown to increase shoot height of dwarf Barley lines, negating the increased stress tolerance exhibited by the dwarf plants (Vettakkorumakankav et al., 1999). SA accumulation has been reported to improve drought tolerance in Arabidopsis by inducing stomatal closure (Khokon et al., 2011) and inhibiting stomatal opening (Okuma et al., 2014). Similarly, in oats, tolerance to drought has been at least partially associated with the accumulation of SA, again by influencing stomatal opening (Sanchez-Martin et al., 2015). However, like for GA, the effect of SA on drought tolerance is complex and others have reported a reduction of drought tolerance by SA application (Miura and Tada, 2014). Since no differential effect on stomatal closure was observed upon progressive drought between the Brachypodium ecotypes, SA may be involved in alternative drought-induced regulatory mechanisms.

An interesting question is how far SA could be influencing the two primary metabolism pathways targeted in our study; the TCA cycle and amino acid metabolism. The TCA cycle is a crucial component of respiratory metabolism and is often altered in plants experiencing stress. Drought induced accumulation of TCA cycle metabolites (Urano et al., 2009) and the upregulation of TCA cycle-related genes in Arabidopsis shoots (Cavalcanti et al., 2014), have been reported. Metabolic differences in the stress tolerance of four different lentil genotypes were related to a reduction in the levels of TCA cycle intermediates suggesting an impaired energy metabolism with consequences on the ability of seedlings to acquire water and to support transport processes (Muscolo et al., 2015). SA has a well-characterized role in maintaining mitochondrial electron flow under stress conditions by inducing the expression of alternative oxidase (AOX) (Feng et al., 2009). This will influence the NADH oxidation by mitochondrial complex I which is coupled to the TCA cycle (Vanlerberghe, 2013). Thus, TOL Brachypodium genotypes could be exhibiting a SA-AOX mechanism of maintaining bioenergetic metabolism during drought.

Amino acid accumulation, in particular proline, is considered a protective mechanism in many water-stressed plants (Rai, 2002). Proline has been shown to increase in several different plant species under drought stress, including maize, wheat, and Miscanthus (Rampino et al., 2006; Witt et al., 2012; Ings et al., 2013), and is thought to function primarily as an osmoprotectant, thereby protecting cells from damage caused by stress (Delauney and Verma, 1993). Indeed, overproduction of proline has been shown to result in increased tolerance to osmotic stress in transgenic plants (Kishor et al., 1995; Zhu et al., 1998; Yamada et al., 2005). The lower levels of both proline and glutamate, which can act as the precursor for proline, in the SUS class, suggests increased sensitivity to drought induced osmotic damage to the ecotypes within this class. In this context, there is evidence suggesting SA can influence the accumulation of some amino acids upon drought stress. A recent study focusing on drought in Creeping Bentgrass (*Agrostis stolonifera*) showed that SA conferred drought tolerance and also the accumulation of proline, serine, threonine and alanine (Li et al., 2016). However, an SA-influenced increased accumulation of carbohydrates, as noted in the Li et al. (2016) study was not prominent in our results.

Despite increasing evidence for the involvement of JA in drought stress, there is little knowledge about its actual role in drought stress signaling, particularly when compared to its involvement in the response to biotic stresses (Du et al., 2013). In some studies, JA seems to improve drought tolerance while in others it has been reported to cause a reduction in growth and yield (Riemann et al., 2015). Interestingly, JA was one of the five metabolites contributing to the discrimination between TOL and SUS/INT under mild stress. A JA-synthesizing lipoxygenase was among the most interacting genes in a regulatory interaction network analyses of Arabidopsis exposed to mild drought stress (Clauw et al., 2015), in agreement with a role of JA in response to mild drought. Higher levels of GA, SA and JA in the TOL Brachypodium ecotypes when exposed to severe drought might suggest a protective role for these hormones. Clearly, cross-talk between these and other hormones (including ABA, ethylene, and auxins) and their associated signal transduction elements may play important roles in the response to drought stress. Further studies are necessary to confirm that increases in GA, SA, and JA are indeed involved in establishing the drought tolerance phenotype exhibited by the TOL Brachypodium ecotypes. In particular, the possible role of JA in influencing primary metabolism to confer drought tolerance in grasses needs to be assessed.

Since analysis of both phenotypic and metabolite data, obtained from the same plant material upon progressive drought, revealed the TOL-SUS-INT clusters, there was an opportunity to assess potential correlations between the two data-sets to suggest metabolic events that could be contributing to the phenotypic changes. Biomass yield and delayed leaf senescence (stay-green) rank among the most important traits for improvement of crop plants under drought stress (Rivero et al., 2007; Salekdeh et al., 2009). Importantly, our analyses identified a number of metabolites (TCA-cycle intermediates, alanine, and SA; as well as JA; see **Supplementary Figure S3**) that correlate positively with phenotypic measures for biomass yield (area and height) and negatively with measures for stress (yellow and grey pixels). These observations would strongly suggest that SA and JA stress hormone signaling is important for maintaining some plant growth and reducing stress phenotypes. Although there is limited biological replication of the metabolite data, such conclusions highlight the value of integrative ‘omic’ approaches in providing novel insights into plant phenomena.

## Conclusion

Drought screens of a diverse Brachypodium ecotype collection, integrated with phenotypic and metabolite profiling of selected Brachypodium ecotypes, highlight the variation in the response of Brachypodium ecotypes to water stress. Combined with its genotypic diversity (Gordon et al., 2014), this confirms the value of Brachypodium as a powerful model for the improvement of cereal, bioenergy, forage, and turf grasses to changing environmental conditions, including drought stress. The combination of phenotypic analysis and metabolite profiling revealed a remarkable consistency in separating the selected ecotypes into their pre-defined drought tolerance groups, highlighting the value of multivariate analysis as a robust approach to analyze complex phenotypic and metabolite data sets. The relative abundance of several metabolites, including for the phytohormones SA and JA, appear to correlate with biomass yield and reduced stress features. Although further studies are necessary to validate these findings, the results highlight the potential advantage of combining the analyses of phenotypic and metabolic responses to identify key mechanisms and markers associated with drought tolerance in crops. Overall, this work shows that different phenotyping assays could reliably identify drought-tolerant and sensitive Brachypodium lines, while metabolite analysis may be predictive. Whilst the current study only provides correlations, future work will be aimed at gaining a better understanding of the genetic basis of the observed differences in the responses to drought stress between selected Brachypodium ecotypes and to investigate causation using recombinant inbred populations (Bettgenhaeuser et al., 2016) and reverse genetics approaches.

## Author Contributions

MB and LM conceived and designed the experiments. LF carried out drought screening and associated analyses and helped with phenotyping. FC and JD supervised plant phenotyping and JH performed image processing and extraction of phenotypic measures. AA performed stomatal conductance measurements. LM supervised metabolite profiling and performed data analyses. TD and KN helped with project design and provided project resources. MB and LM wrote the manuscript and LF, FC, TD, KN, and JD provided critical comments for manuscript improvement. All authors have read the manuscript and agree with its content.

## Conflict of Interest Statement

The authors declare that the research was conducted in the absence of any commercial or financial relationships that could be construed as a potential conflict of interest.

## Footnotes

1 http://www.opencv.org/

2 http://www.genome.jp/kegg/

3 http://maltese.dbs.aber.ac.uk:8888/hrmet/index.html

4 http://jgi.doe.gov/our-science/science-programs/plant-genomics/brachypodium/

## Supplementary Material

FIGURE S1 **Stomatal performance in selected Brachypodium ecotypes.** At 5 weeks old, the nine Brachypodium accessions were either watered on a daily basis or droughted through non-watering. Stomatal conductance was determined at mid-point in the light period for each plant using a porometer every other day. Results are presented as mean conductance (*n* = 5 plants ± SE). Results are presented in three registers corresponding to susceptible (SUS), intermediate (INT) and tolerant (TOL) genotypes, respectively.

FIGURE S2 **Brachypodium ecotypes 12 days after initiation of treatments (45 das).** Side image photographs show two out of the four plants harvested for metabolite profiling for each of the ecotypes. TOL, tolerant; INT, intermediate; SUS, susceptible.

FIGURE S3 **Correlation analyses of phenotypic and metabolomic data.** Metabolites tentatively linked to four key pathways as targeted by unbiased chemometric analyses were correlated with phenotypic datasets. The degree of Pearson’s correlation is indicated as the intensity of red (positive) or green (negative) color. *r*^2^ values are provided in **Supplementary Table S4**.

TABLE S1 **Overview of the Brachypodium distachyon germplasm collection used for the two drought screens.** Origin and further geographical information available is listed for each of the 138 ecotypes. All accession are from IBERS collection, except ^∗^ Benavente Collection; ^∗∗^ ABR collection; ˆ Garvin collection; ˆˆ Vogel collection. Ecotypes included in second drought screen are highlighted in grey.

TABLE S2 **Mean wilting scores obtained for each ecotype included in first drought screen.** Scores are averages of three individual plants except ^∗^, based on two plants. Ecotypes included in second drought screen are highlighted in grey. SD, standard deviation.

TABLE S3 **Normalized shoot area data.** Normalized shoot area data, derived from side images, used to prepare graphs shown in **Figure 3**. Standard deviation of the measurements is included (SD). Abbreviations: das, days after sowing; S-area, side area; SWC, soil water content.

TABLE S4 **Multivariate regression analyses (*R*^2^) of phenomic and metabolomics data**.
