# Supplementary material for: Linking Dynamic Phenotyping with Metabolite Analysis to Study Natural Variation in Drought Responses of Brachypodium distachyon
Source: Front Plant Sci. 2016 Nov 29;7:1751. doi: 10.3389/fpls.2016.01751 (PMC5126067; doi:10.3389/fpls.2016.01751)
Supplement: FIGURE S1 — Stomatal performance in selected Brachypodium ecotypes. At 5 weeks old, the nine Brachypodium accessions were either watered on a daily basis or droughted through non-watering. Stomatal conductance was determined at mid-point in the light period for each plant using a porometer every other day. Results are presented as mean conductance (n = 5 plants ± SE). Results are presented in three registers corresponding to susceptible (SUS), intermediate (INT) and tolerant (TOL) genotypes, respectively. [file Presentation_1.PPTX]

## Slide 1
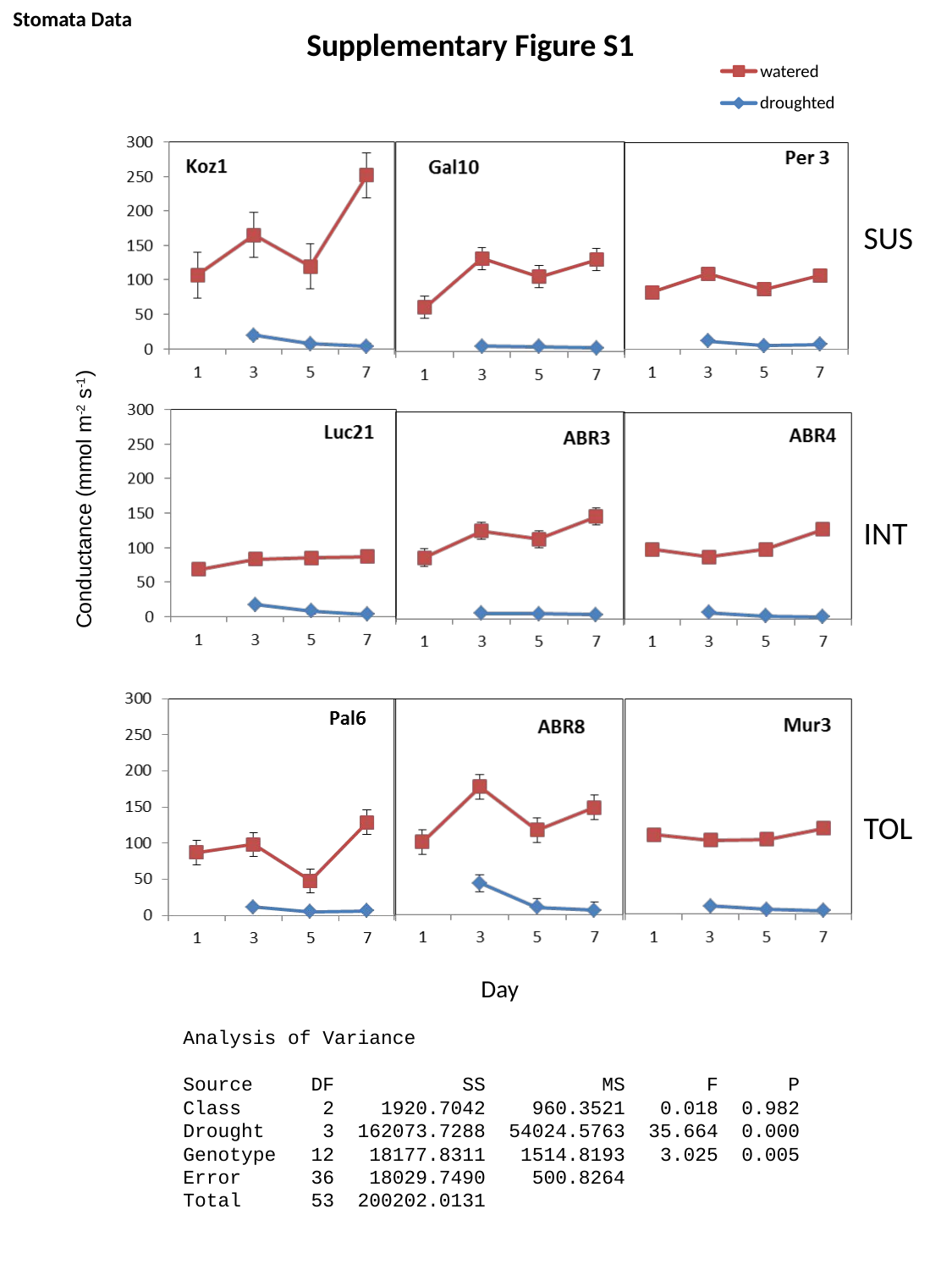

Stomata Data
Supplementary Figure S1
watered
droughted
SUS
Conductance (mmol m-2 s-1)
INT
TOL
Day
Analysis of Variance
Source DF SS MS F P
Class 2 1920.7042 960.3521 0.018 0.982
Drought 3 162073.7288 54024.5763 35.664 0.000
Genotype 12 18177.8311 1514.8193 3.025 0.005
Error 36 18029.7490 500.8264
Total 53 200202.0131

## Slide 2
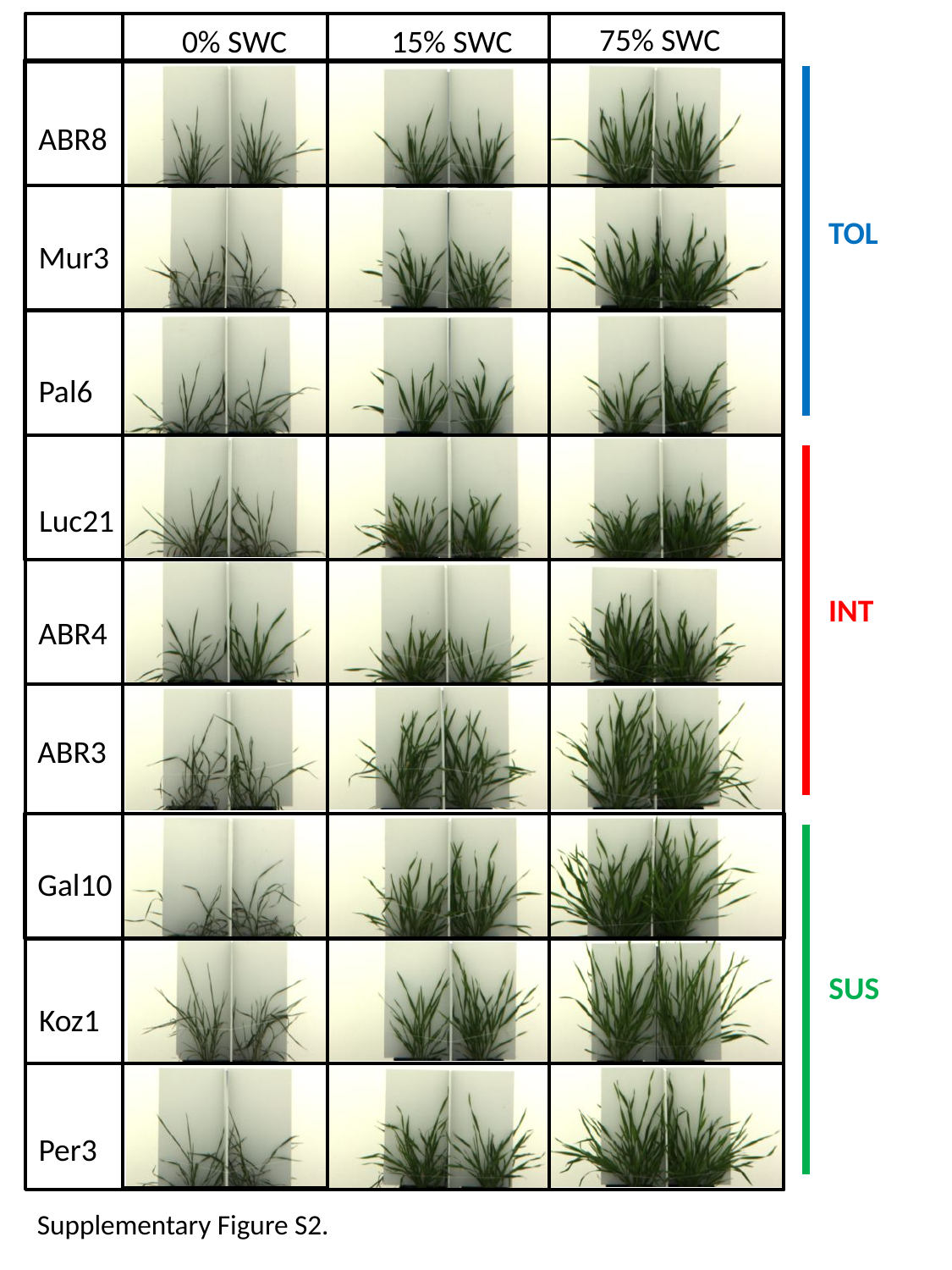

75% SWC
0% SWC
15% SWC
ABR8
TOL
Mur3
Pal6
Luc21
INT
ABR4
ABR3
Gal10
SUS
Koz1
Per3
Supplementary Figure S2.

## Slide 3
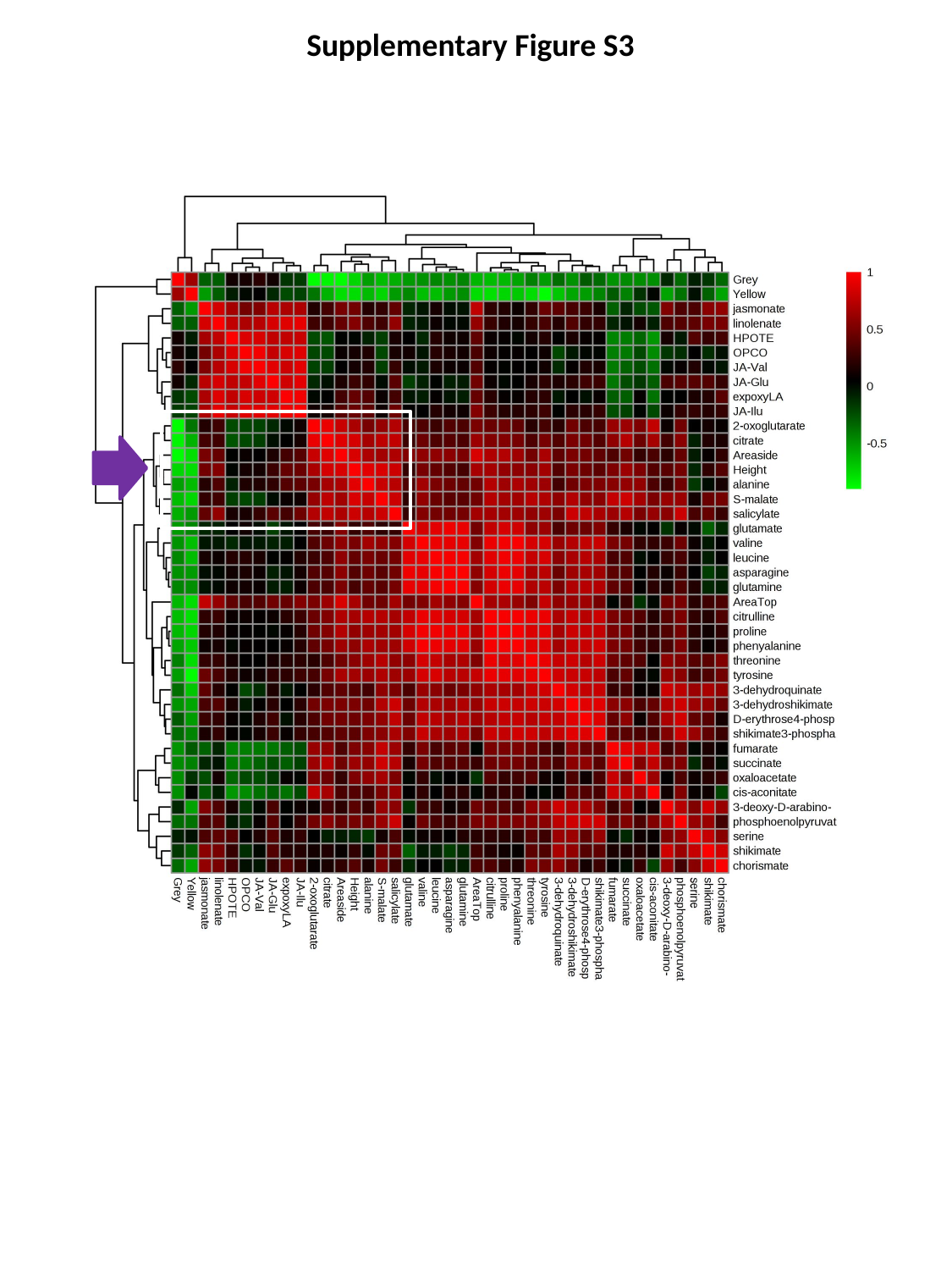

Supplementary Figure S3
