# Supplementary material for: Linking Dynamic Phenotyping with Metabolite Analysis to Study Natural Variation in Drought Responses of Brachypodium distachyon
Source: Front Plant Sci. 2016 Nov 29;7:1751. doi: 10.3389/fpls.2016.01751 (PMC5126067; doi:10.3389/fpls.2016.01751)
Supplement: TABLE S4 — Multivariate regression analyses (R2) of phenomic and metabolomics data. [file Table_4.DOC]

Supplementary Table S4: Multivariate regression analyses (R^2^) of phenomic and metabolomics data

|  | Grey |  | | |  | |  | |  | |  | |  | |  | |  | |  |  |
| --- | --- | --- | --- | --- | --- | --- | --- | --- | --- | --- | --- | --- | --- | --- | --- | --- | --- | --- | --- | --- |
| Grey | 1 | | Yellow | |  | |  | |  | |  | |  | |  | |  | |  |  |
| Yellow | 0.64182 | | 1 | fumarate | | |  | |  | |  | |  | |  | |  | |  |  |
| fumarate | -0.49947 | | -0.28541 | 1 | | Area Top | |  | | |  | |  | |  | |  | |  |  |
| AreaTop | -0.62649 | | -0.76499 | 0.036666 | | 1 | | Area side | |  | | |  | |  | |  | |  |  |
| Areaside | -0.89511 | | -0.77475 | 0.34378 | | 0.85316 | | 1 | | 2-oxoglutarate | | | | |  | |  | |  |  |
| 2-oxoglutarate | -0.89225 | | -0.37847 | 0.61775 | | 0.50701 | | 0.79842 | | 1 | | citrate | |  | | |  | |  |  |
| citrate | -0.8564 | | -0.61332 | 0.59262 | | 0.63682 | | 0.88763 | | 0.92409 | | 1 | | alanine | |  | | |  |  |
| alanine | -0.53794 | | -0.64234 | 0.56108 | | 0.47959 | | 0.69241 | | 0.49733 | | 0.67231 | | 1 | | malate |  | | |  |
| malate | -0.64952 | | -0.74674 | 0.78484 | | 0.47349 | | 0.6522 | | 0.61207 | | 0.75863 | | 0.79961 | | 1 | Height |  | | |
| Height | -0.74155 | | -0.76894 | 0.52417 | | 0.67538 | | 0.85753 | | 0.68851 | | 0.84548 | | 0.89091 | | 0.86308 | 1 | salicylate | | |
| salicylate | -0.59841 | | -0.51908 | 0.63524 | | 0.65502 | | 0.69939 | | 0.71474 | | 0.77421 | | 0.73815 | | 0.80957 | 0.81221 | 1 | | |
